# Molecular epidemiology of *Cryptosporidium species* in Kpong and its environs, Ghana

**DOI:** 10.1371/journal.pone.0281216

**Published:** 2023-02-24

**Authors:** George T. Mensah, Patrick F. Ayeh-Kumi, Abraham K. Annang, Isaac Owusu-Frimpong, Sena Niampoma, Charles A. Brown

**Affiliations:** 1 Environmental Biology, Health, and Biotechnology Division, CSIR- Water Research Institute, Accra, Ghana; 2 Department of Microbiology, School of Biomedical and Allied Health Sciences, College of Health Sciences, University of Ghana, Korle-Bu, Ghana; 3 Parasitology Department, Noguchi Memorial Institute for Medical Research, College of Health Sciences, University of Ghana, Legon, Ghana; 4 Biomedical and Public Health Research Unit, CSIR–Water Research Institute, Accra, Ghana; 5 Department of Nutrition and Dietetics, School of Biomedical and Allied Health Sciences, College of Health Sciences, University of Ghana, Korle-Bu, Ghana; King Abdulaziz City for Science and Technology (KACST), SAUDI ARABIA

## Abstract

**Background:**

*Cryptosporidium* is a ubiquitous enteric protozoan pathogen infecting humans, domestic animals, and wildlife worldwide. It is a waterborne pathogen with recognized zoonotic potential and a definite cause of diarrhea and nutritional disorders in institutional and community settings. One challenge facing the world’s supply of clean drinking water is contamination from feces and soil. It has been established that small quantities of oocysts, the infective stage, can cause human disease. Also, their resistance to chlorination and other water treatment procedures has been demonstrated. Kpong, a community in the Lower Manya Krobo Municipality of the Eastern Region of Ghana, is one of the primary sources of water supply to Accra, the capital city of Ghana. Being able to determine the effectiveness of water treatment processes and identifying sources of contamination of this pathogen in our water bodies is thus of public health importance. The study aimed to conduct molecular epidemiology of *Cryptosporidium* spp. in the Lower Manya Krobo Municipality.

**Methodology/Principal findings:**

A total of 230 samples, 180 fecal samples from cattle and 50 water samples (tap water and well water) were collected from the following communities: Kpong, Akwernor, Ablotsi, Nuaso, and Atua, all in the Lower Manya Krobo Municipality. Samples were screened for *Cryptosporidium* by microscopy and PCR. The 18S rRNA gene was amplified by nested polymerase chain reaction (PCR), and the final product was sequenced. The prevalence of *Cryptosporidium* from the fecal samples was estimated as 10% (18/180) by microscopy, while all 50 water samples were negative. However, PCR gave the prevalence of *Cryptosporidium* as 47.8% (86/180) for fecal samples and 20% (10/50) for water samples. Based on the 18S rRNA gene, three sequenced samples showed high homology to *C*. *parvum* species. The phylogenetic analysis confirmed this as these sequences clustered with *C*. *parvum* sequences from other countries.

**Conclusion/Significance:**

*Cryptosporidium parvum* was identified as the persistent species in the study communities. This outcome supports the evidence that domesticated animals serve as potential reservoirs of zoonotic transmission of cryptosporidiosis. The persistence of cryptosporidiosis in cattle indicates its presence in the human population. In addition, the presence of *Cryptosporidium parvum* in the wells makes it alarming and necessary to consider a holistic approach such as One Health Strategies to identify and control cases in humans.

## Introduction

Cryptosporidiosis is a parasitic disease caused by *Cryptosporidium species*, a protozoa parasite that emerged to be responsible for diarrhea in humans and domesticated animals [1–6]. Since *Cryptosporidium* affects a wide range of vertebrate hosts, including humans and animals, it is considered a zoonotic parasite [7–9]. Cryptosporidiosis is a parasitic-enteric disease responsible for morbidity and mortality in young calves [10–12]. It is also known to be responsible for causing harm to immunocompromised humans [13]. This disease presents with diarrhea, abdominal pain, fever, nausea, vomiting, and weight loss [14]. Water and food are known to be the main vehicles of transmission of this parasite since transmission is by the fecal-oral route [15]. It has been established that *Cryptosporidium* spp. can be found in market vegetables, drinking source water, coastal water, recreational use water, and wastewater [16].

Cryptosporidiosis has been reported in humans in more than 90 countries on all continents except Antarctica [17]. Different *Cryptosporidium* species have been documented in Europe, the Americas, Australia, and Africa, with the predominant species being *C*. *hominis* and *C*. *parvum* [18, 19]. These organisms are more common in surface water than in groundwater because surface water is easier to be contaminated with water from sewage discharge [20–22]. At least 44 *Cryptosporidium* species have been described, and around 120 different genotypes have been identified through genetic characterization [16]. The application of molecular techniques has been instrumental in describing host-specific species and those capable of infecting multiple hosts [23]. It is now easier to study the epidemiology of cryptosporidiosis using molecular methods for the genetic characterization of *Cryptosporidium* sp. [24].

Many diagnostic methods, including microscopy, immunological assays, and molecular techniques, have been established to detect *Cryptosporidium* spp. [25, 26]. Microscopy, the commonly used diagnostic approach, involves the morphological identification of oocysts in fecal samples. To achieve this, a wet mount or stain such as Acid Fast, Lugol’s iodine, or an immunofluorescent antibody stain is used [25, 26]. Also, fecal-antigen diagnostic approaches such as enzyme immunoassays (EIA), enzyme-linked immunosorbent assays (ELISA), and immunochromatographic tests (ICT) were developed to maximize accuracy and reduce test time [26, 27]. However, immunological assays have been reported to generate false-positive results [28, 29]. Polymerase chain reaction (PCR) evolved to resolve the limitations observed in the other diagnostic methods. Since PCR allows for batch testing, species, and sub-species identification of discovered organisms, it is more sensitive than traditional microscopy and immunological approaches for detecting *Cryptosporidium* [30–32].

Globally, numerous reports of cattle and other livestock infected with *Cryptosporidium* spp. have been established [23, 33–38]. For example, in Nigeria, numerous investigations of livestock illnesses with *Cryptosporidium* have been carried out [39–42]. On the other hand, few studies have been conducted in Ghana on *Cryptosporidium* in cattle and other livestock, despite reported prevalence rates of more than 19% [43–46].

Kpong has a water treatment plant that supplies water to about 2.5 million people in the Greater Accra Region. Upstream this treatment plant, are dotted cattle farms which can be a potential source of contamination of the river with this parasite, and this can bring an epidemic should the fecal matter from these farms, which had been found to contain this parasite find its way to the river. In addition, studies have established that conventional water treatment methods do not eliminate this parasite from portable water production when water sources are contaminated [47, 48]. Furthermore, with the zoonotic potential of this parasite well established, it is, therefore, necessary that we protect water bodies to prevent its spread into the rivers used for potable water production and other sources of water for domestic use. This study determined the prevalence of this parasite (*Cryptosporidium* spp.) and identified the various species present at Kpong and its environs. Furthermore, based on their 18S small-subunit rRNA genes, the phylogenetic relationships of the *Cryptosporidium* isolates were also determined.

## Materials and methods

### Ethics statement

Ethical approval for the study was obtained from the Institutional Review Board of the Council for Scientific and Industrial Research (CSIR) with reference RPN 005/CSIR-IRB/2013.

### Study area

**Fig 1:**
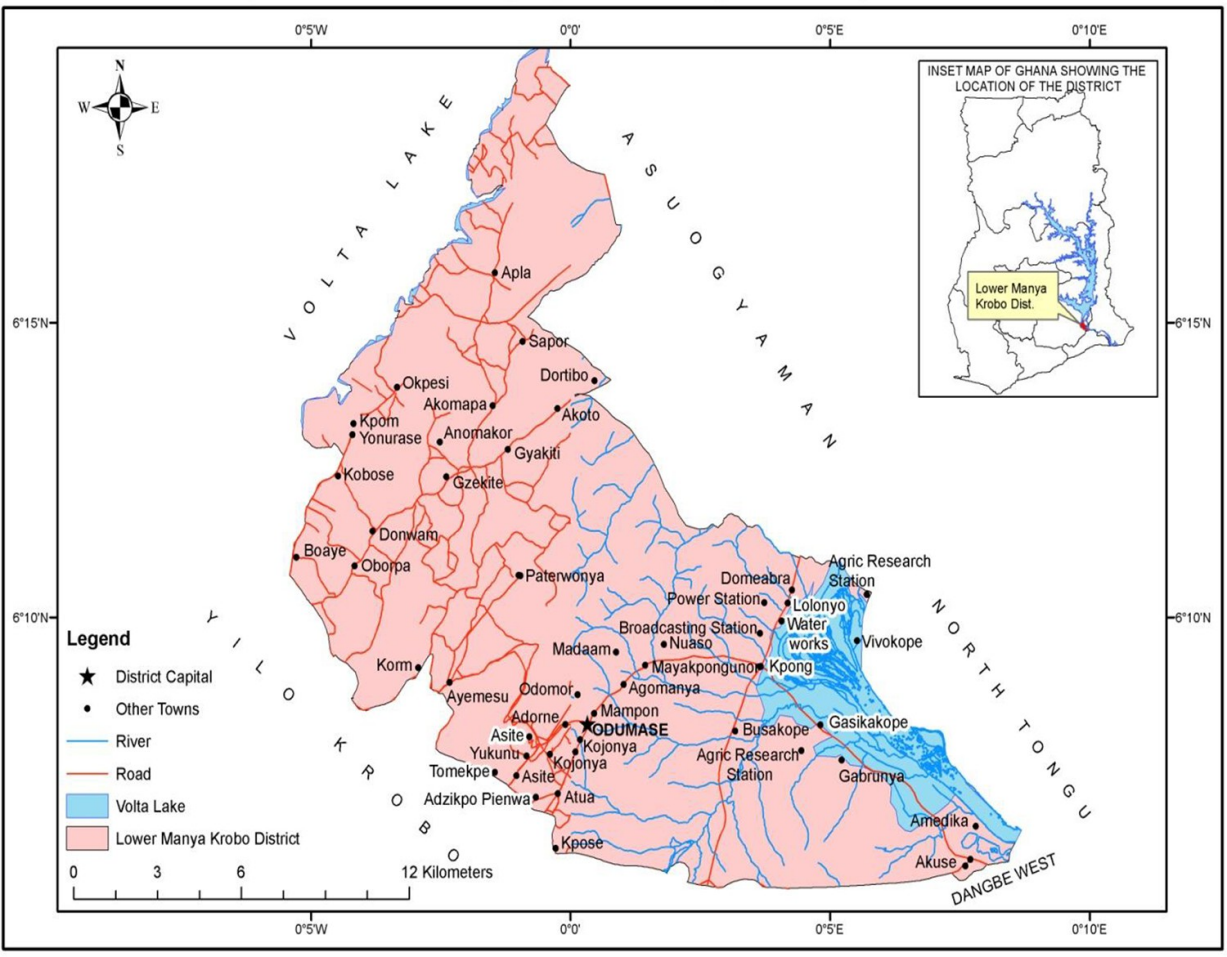
Map of the lower Manya Krobo municipal area showing the selected communities. (Source: adapted from the previous study conducted by Mensah and colleagues [43]).

Kpong and four selected communities, Akwernor, Ablotsi, Nuaso, and Atua, in the Lower Manya Krobo Municipality (Fig 1), were used for the study. The Lower Manya Kobo Municipality is one of the 26 Metropolitan, Municipal and District Assemblies (MMDA) in the Eastern Region of Ghana. The administrative capital of the Municipality is Odumase-Krobo. The Municipality covers an area of 304.4 sq km (Ghana Statistical Service, 2014). The major towns in the Municipality include Odumase township (which incorporates Atua, Agormanya, and Nuaso), Akuse, and Kpong in the Lower Manya area (Ghana Statistical Service, 2014). Kpong Hydro Electric Project and the resultant dam are sited in this Municipality. In addition, the Kpong water treatment plant supplies water to most parts of the Greater Accra Region of Ghana. Three cattle farms were used for this study, and each farm had about 70–100 animals made up of calves, weaned calves, and adults. The farms were in the village community of Kpong with about 30 households, each having between 5–10 persons. The children in the community were about 30 in number and mostly below the age of five (5) years.

### Sample collection and study design

This study was a field-based cross-sectional study carried out for nine months from October 2013 -June 2014. A total of 180 fecal samples from cattle of varying ages and 50 water samples (25 tap water and 25 well water) were used for the study. Fresh fecal samples were collected from three cattle farms upstream of the Kpong water treatment plant. In addition, water samples were collected from the water treatment plant laboratory, taps, and wells from all the study sites.

The fecal samples were collected as soon as the animals egested onto the ground using disposable wooden spatulas. Care was taken to avoid environmental contamination by sampling only those portions of the fecal material that had no contact with the ground. About 30g of stool was collected into a labeled sterile wide-mouth stool container with lids. The fecal samples were stored on ice and transported to the laboratory for processing and analysis within six (6) hours. In addition, 5-liter grab water samples were collected each month from the taps and wells in each of the five communities. Up to 240 liters of water was also sampled at the water treatment works.

### Laboratory analysis

#### Processing of fecal samples

Five grams of each fecal specimen was placed into two 15ml falcon tubes; one tube contained 10 ml phosphate-buffered saline (PBS) and the other 10 ml isopropanol. Each tube was vortexed and sieved through cotton gauze to remove large particles. The mixture in the isopropanol was stored in a refrigerator (4°C) for use in molecular analysis. The sample in PBS was centrifuged at 10,000 rpm for 5 minutes, and the supernatant was used for the microscopy [49].

#### Microscopy

For *Cryptosporidium* sp. oocyst identification, the supernatant was stained using the Modified Ziehl Neelsen technique [50, 51]. First, the supernatant was smeared on a microscope slide using a sterilized loop. Next, it was fixed with methanol for 20 minutes, stained with carbol-fuchsin, destained with 10% sulphuric acid for 5 minutes each, and washed under running tap water for 5 minutes. Finally, it was counter-stained with methylene blue for 30 seconds, washed under running tap water for 5 minutes, drained, and air-dried.

#### DNA extraction

DNA was extracted from the water and fecal samples in isopropanol using QIAamp DNA Stool Mini Kit (Qiagen Inc., USA) according to the manufacturer’s protocol. Before DNA extraction, samples were thoroughly mixed and 200 μl suspended in 1.4 ml Buffer ASL (lysis buffer). Next, the oocysts were ruptured by putting them through three cycles of freezing at -40°C and thawing in an incubator at 90°C. DNA samples were later quantified with JENWAY Genova Plus Spectrophotometer (Bibby Scientific Ltd., UK) and stored at -20°C until ready for use.

#### Cryptosporidiosis detection with nested 18S rRNA

*Cryptosporidium sp*. presence was further detected in the DNA samples with nested PCR to amplify a 465bp fragment from the18S rRNA gene, as described by Nichols and colleagues [52]. The initial reaction (Nest 1) involved the use of the external forward primer OutN_DIAGF2 (5ʹ-CAATTGGAGGGCAAGTCTGGTGCCAGC-3ʹ) and external reverse primer OutN_DIAGR2 (5ʹ -CCTTCCTATGTCTGGACCTGGTGAGT-3ʹ) to amplify a 655–667 bp fragment. The PCR reaction mix involved a total of 25 μL mixture which included 5 μL DNA template, 1x DreamTaq Buffer, dNTP mix (0.2 mM of each of dATP, dTTP, dCTP, and dGTP), 200nM each of forward and reverse primers and 1.25U DreamTaq DNA Polymerase (Thermo Fisher Scientific Inc., UK). The cycling condition consisted of an initial denaturation step of 3 mins at 94°C followed by 30 cycles of 30s at 94°C, 60s at 55°C, and 45s at 72°C. The final extension was performed at 72°C for 10 mins. The PCR reaction was performed with the Applied Biosystem 2720 thermal cycler (Applied Biosystem, USA), with positive and negative controls included as a quality control check. About 10 μL of Nest 1 PCR products were run on 2.0% agarose gel, stained with 0.5 μg/mL ethidium bromide, and visualized under UV light (Kodak EDAS 290 gel documentation system) to confirm the expected 655–667 bp target.

The secondary PCR (Nest 2) assay was performed to amplify a 465 bp fragment using internal forward primer InDIAGF (5ʹ-AAGCTCGGTAGTTGGATTTCTG-3ʹ) and internal reverse primer InDIAGR (5ʹ-TAAGGTGCTGAAGGAGTAAGG-3ʹ). A total of 25 μL reaction mixture which consists of 1 μL Nest 1 PCR product, 1x DreamTaq Buffer, dNTP mix (0.2 mM of each of dATP, dTTP, dCTP, and dGTP), 200nM each of forward and reverse primers and 1.25U DreamTaq DNA Polymerase (Thermo Fisher Scientific Inc., UK). The program consisted of an initial denaturation step of 3 mins at 94°C followed by 30 cycles of 30s at 94°C, 30s at 58°C, and 30s at 72°C. The final extension was performed at 72°C for 10 mins. The Applied Biosystem 2720 thermal cycler (Applied Biosystem, USA) was used for this reaction. Nest 1 positive and negative (non-template) controls were used for the Nest 2 reaction as a quality control check. Afterward, the Next 2 PCR products were electrophoresed on 2.0% agarose gel, stained with 0.5 μg/mL ethidium bromide, and visualized under UV light (Kodak EDAS 290 gel documentation system) to confirm the expected 465 bp target.

#### DNA sequencing

Commercial sequencing of the PCR products of *Cryptosporidium sp*. samples was done at the Noguchi Memorial Institute for Medical Research, University of Ghana, Legon. Sequence analysis was performed by first editing the sequence data with Bioedit Sequence Alignment Editor (version 7.2.5) software. Afterward, sequences were compared with published sequences in the GenBank using the Basic Local Alignment Search Tool (BLAST) from the National Center for Biotechnology Information (NCBI) (http://blast.ncbi.nlm.nih.gov/Blast.cgi) to determine closely related sequences. Sequences of interest were later acquired and aligned to determine the maximum homology.

Phylogenetic and molecular evolutionary analyses were conducted on our sequenced data and the homologous sequences using MEGA version 6 [53]. The sequence data were aligned using the CLUSTAL W package [54] integrated into the MEGA 6 software package. Parameters used for both pairwise and multiple alignments include an open gap penalty of 30 and an extended gap penalty of 10. Also, a slow alignment mode was selected for pairwise alignments. A delay divergence of 60% was selected for multiple alignments, and the transitions were weighted. The Maximum Likelihood algorithm based on the Tamura-Nei model for inferring phylogeny was used to evaluate the sequence relationships. Automatical Initial tree(s) for the heuristic search were obtained by applying Neighbor-Join and BioNJ algorithms to a matrix of pairwise distances estimated using the Maximum Composite Likelihood (MCL) approach and then selecting the topology with a superior log likelihood value. The tree is drawn to scale, with branch lengths measured in the number of substitutions per site. The analysis involved 38 nucleotide sequences. Codon positions included were 1st+2nd+3rd+Noncoding. All positions containing gaps and missing data were eliminated. There was a total of 360 positions in the final dataset.

### Statistical analysis

Statistical analysis was performed with GraphPad Prism version 8.0 (GraphPad Software, Inc., San Diego, CA, USA; https://www.graphpad.com) for the descriptive statistical analysis, Chi-square statistics, and the prevalence of *Cryptosporidium sp*. across various age groups. A confidence level of 95% was set, and *p*<0.05 was considered statistically significant.

## Results

### Sample description

Of the 230 samples, 180 were fecal samples from cattle, and 50 water samples from the tap (25) and wells (25) were collected for the study. Fecal samples were collected at different time points within the study period and more samples were collected from adult cattle compared to calves and weaned calves [Table 1].

**Table 1:** Fecal samples collected per visit according to the cattle age groups.

Month	Age group			Total
	Calves	Weaned calves	Adults	
**October**	6	6	18	30
**December**	6	6	18	30
**February**	6	6	18	30
**April**	9	9	27	45
**June**	9	9	27	45
**Total**	**36**	**36**	**108**	**180**

### *Cryptosporidium* infection status

**Fig 2:**
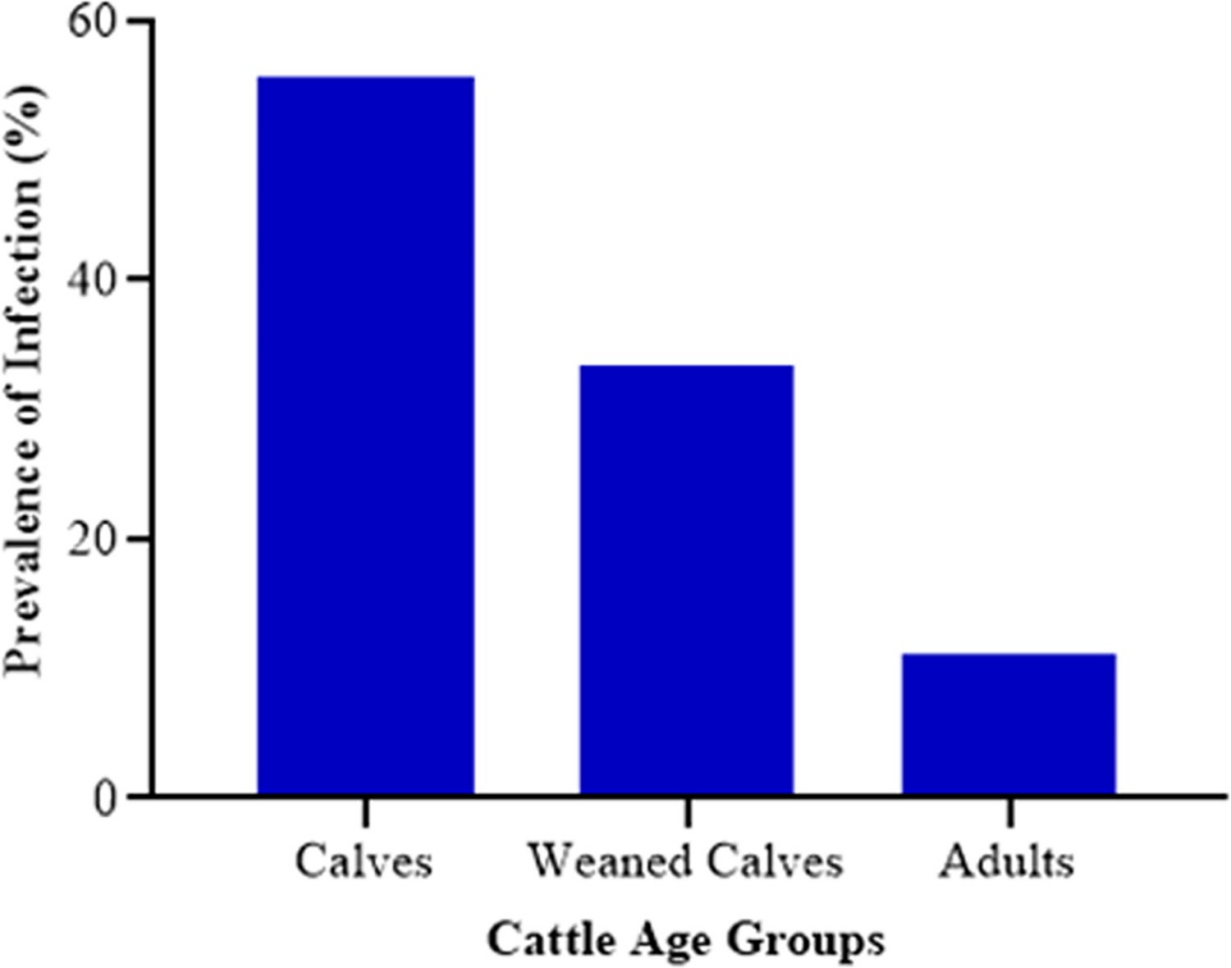
Distribution of *Cryptosporidium sp*. among the cattle age-group.

Out of the 180 fecal samples analyzed by microscopy, 18 (10%) were positive for *Cryptosporidium sp*. Conversely, the 50 water samples were negative for detecting *Cryptosporidium sp*. by microscopy. The calves had the highest number (55.56%) of positives out of the 18 identified (Fig 2).

### Other parasitic infections

**Fig 3:**
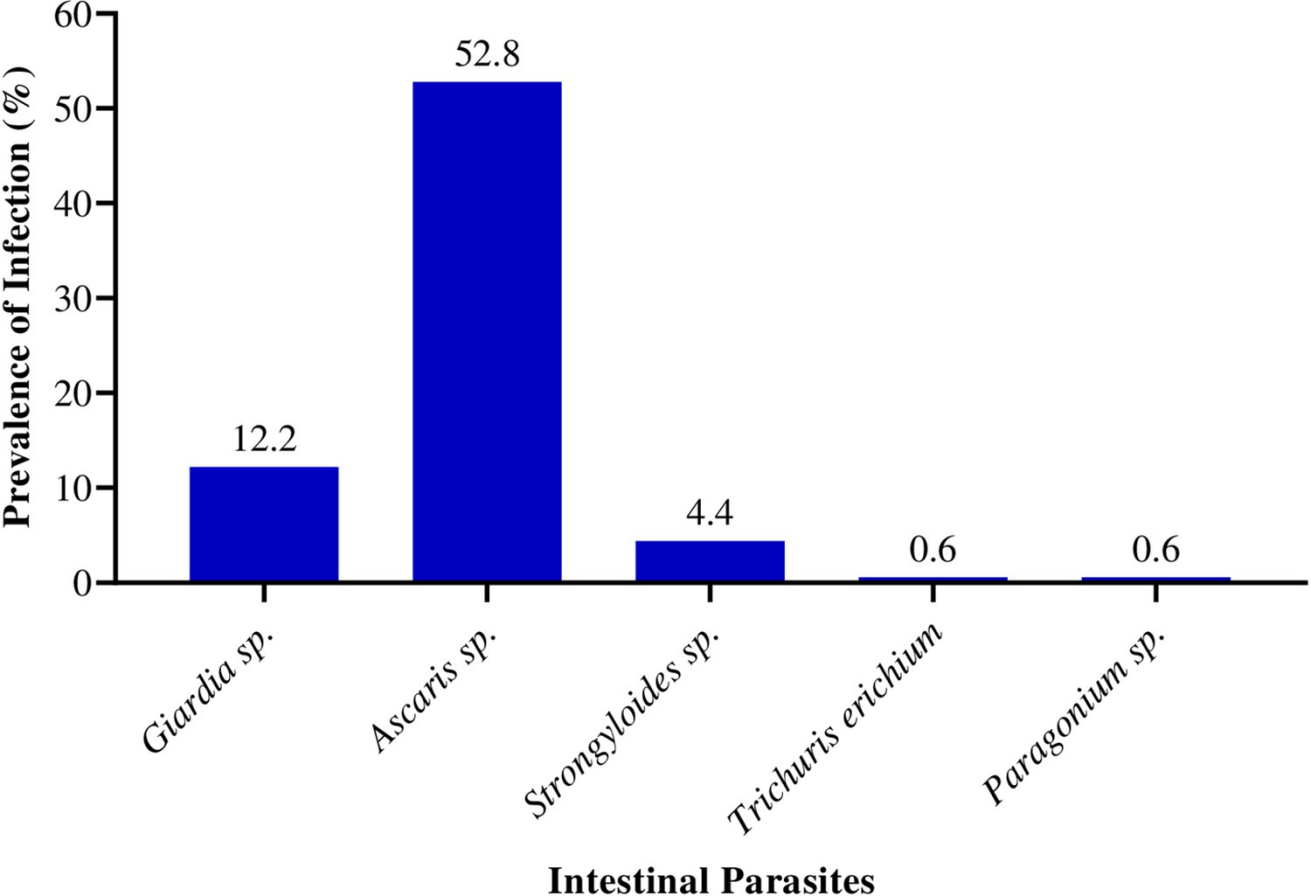
Prevalence of other intestinal parasites.

Other parasitic infection were identified by microscopy in the 180 fecal samples with *Ascaris sp*. being the highest (52.8%) and *Trichuris erichium* and *Paragonimus sp*. being the least, (0.6%) each (Fig 3). Also, the study saw a few cases of mixed infections; 5 (2.8%) samples had mixed infections of *Ascaris sp*. and *Strongyloides sp*., and 1 (0.6%) sample had a mixture of *Ascaris sp*. and *T*. *erichiun*.

### Molecular analysis

Of the 180 fecal samples screened for the presence of *Cryptosporidium* DNA using nested PCR, 86 (47.8%) were positive. Also, nested PCR was positive for 10 (20%) of the water samples. However, all these were well water samples collected during each study month only in Atua and Akwenor.

Six PCR products obtained from the nested PCR were sequenced. However, three (3) gave poor-quality reads and were therefore rejected. The three (3) successfully sequenced samples, 62_CAin, 92_CAin, and 102_Cain, were identified as *Cryptosporidium* DNA sequences (see S1 Fig. Figs 1–3). Also, the comparative analysis of the sequenced data showed that the sequences exhibited 95–97% similarity (Table 2).

**Table 2:** Overall similarities between the *Cryptosporidium* DNA sequences.

Sequence->	% Similarity		
	62_CAin	92_CAin	102_CAin
**62_CAin**	ID	97.4	97.0
**92_CAin**	97.4	ID	95.4
**102_CAin**	97.0	95.4	ID

### Phylogenetic analysis

**Fig 4:**
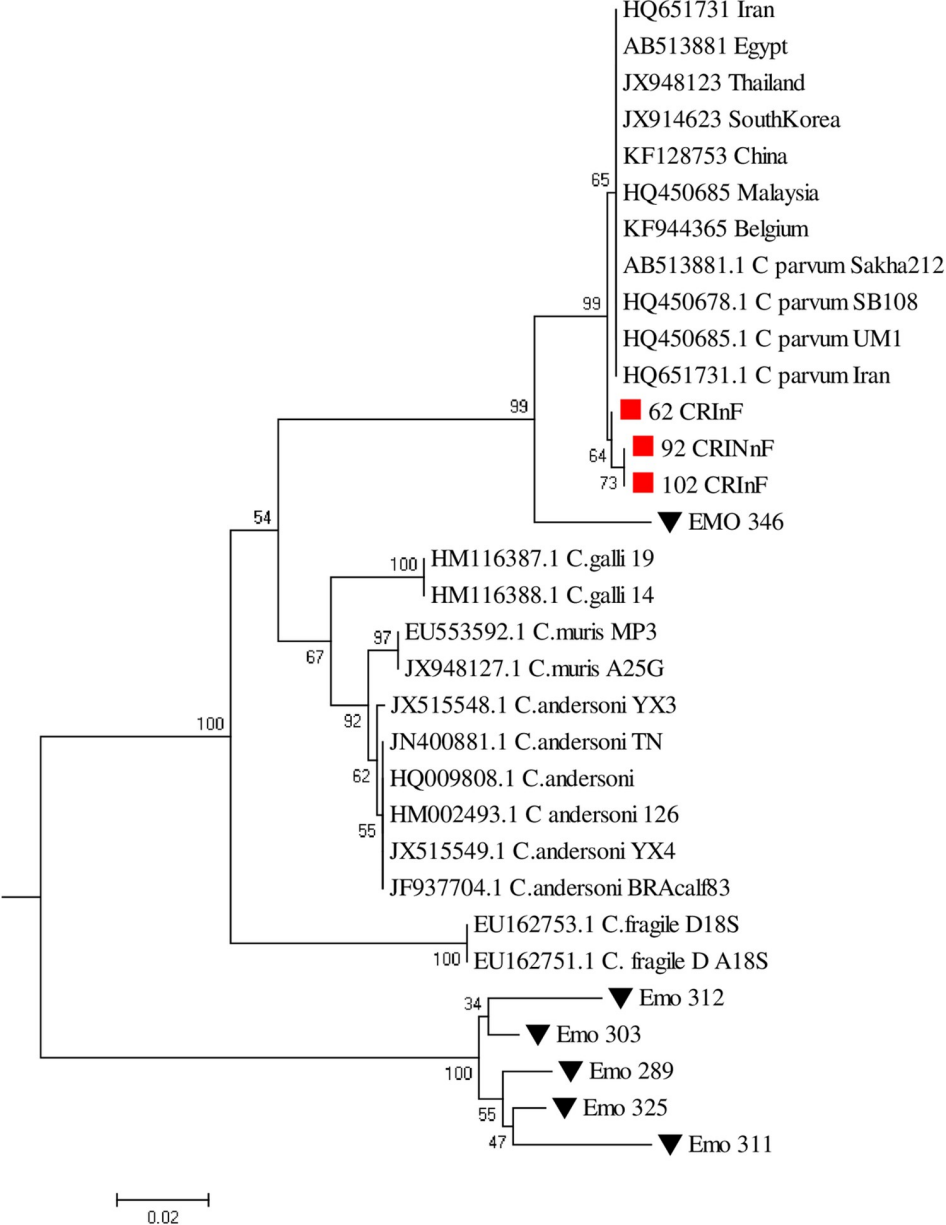
Molecular phylogenetic analysis by maximum likelihood method. The tree with the highest log likelihood (-1334.4013) is shown. The percentage of trees in which the associated taxa clustered together is displayed next to the branches. Red square Sequences obtained from this study. Black triangle Sequences obtained from a study in Accra, Ghana (Anim-Baidoo [55]).

The phylogenetic tree was based on the results of distance matrix analysis of 62_CAin, 92_CAin, and 102_CAin to the homologous 18S rDNA sequences. It was clear from both the tree topology and distance consideration that all three sequences have a close phylogenetic affinity with *C*. *parvum* (Fig 4). Furthermore, bootstrap analysis data (bootstrap value >50) confirmed that this association was highly significant (*p*< 0.05).

## Discussion

Diarrheal diseases are common among infants in developing countries, including Ghana, with a significant cause of morbidity and mortality [10–13]. *Cryptosporidium sp*. is a vital parasite associated with diarrhea in communities where domesticated animals coexist with humans due to its zoonotic potential [1–3, 56]. In addition, the disease has been associated with poor sanitation and contamination of potable water and other water sources available for domestic chores [7, 20–22]. The present study aimed to determine the presence of *Cryptosporidium* sp. in cattle farms near the Kpong and its environs.

In this study, the overall prevalence rates obtained from the molecular analysis of fecal and water samples for detecting *Cryptosporidium sp*. in cattle and well water sources were 47.8% and 10%, respectively. This outcome proves the presence of the pathogen in the selected communities, more importantly, in the cattle-rearing areas and some wells at various distances from the cattle kraals. These wells may have been contaminated by fecal matter from the cattle kraals through run-off water, human activities, or using the same water drawing containers for cattle and humans.

The outcome of this study was found to be higher than previous studies conducted on cattle in Ghana. Firstly, a study conducted among cattle in the Central region of Ghana recorded a prevalence of 23.7% after the application of antigen-detecting ELISA [46]. In the same way, our prevalence was higher than that of an earlier study in the Kpong area (23.8%) after screening with the Ziehl-Neelsen modified acid-fast staining technique [1]. In addition, another study conducted in the Greater Accra region applied the use of the Ziehl-Neelsen modified acid-fast staining technique as a diagnostic tool for screening cattle and reported a prevalence of 29%, which is lower than the one reported in this study [44]. Furthermore, another study conducted in three regions in Ghana (Greater Accra, Central, and Volta regions) reported an overall prevalence of *Cryptosporidium sp*. in livestock as 30.8% after applying quantitative PCR [45]. However, the prevalence in cattle was recorded as 26.5%, which is lower than that reported in this study.

Comparing the prevalence of this study to studies in other parts of the world showed a good relationship. For instance, in Nigeria, the prevalence of *Cryptosporidium sp*. in cattle has been reported as 23.4% and 37.5% after using Kinyoun acid-fast staining and coproantigens methods, respectively [40, 41]. Also, another study conducted in Ethiopia which applied both modified Ziehl-Neelsen staining and molecular techniques, arrived at a prevalence of 18.6% [33]. Another study in Turkey reported a prevalence of cryptosporidiosis as 27.4% in calves after molecular diagnosis [37]. In China, a study conducted on pre-weaned dairy calves after screening for C*ryptosporidium* by PCR reported a prevalence of 47.68%, similar to that reported in this study [38]. Numerous factors, such as the sampling season, the geographic and ecological location of the study areas, the laboratory techniques used to detect the parasite, the type of animal management system used by the farmers, the breed of the animals, the state of hygiene of the farms or kraals, as well as the climate of the area under study, may contribute to the varying prevalence of *Cryptosporidium* infection among cattle observed in different studies [44, 46].

This study also showed that the parasite affected more calves and weaned calves than adults, which can be reservoirs for environmental contamination if not controlled [11, 44, 45, 56]. This observation made is due to the suppressed immunity in juveniles. Squire and colleagues made this same observation in Ghana among ruminants [45]. Furthermore, they demonstrated that the prevalence of cryptosporidiosis was higher in the younger age group than in adults; thus, the risk of infection decreased as the cattle aged. Also, Mueller-Doblies and colleagues reported that the young age group was more responsible for initiating zoonotic transmissions than the adults [57]. Finally, a study on cats in Australia ascertained that the disease occurs far more commonly in immune-compromised animals and humans than in immune-competent ones [58]. This conclusion was because they observed the same trend where the *C*. *parvum* was more prevalent in kittens than the adults.

The genetic sequencing and phylogenetic analysis confirmed *Cryptosporidium parvum* as the species present in the study areas as they clustered with *C*. *parvum* sequences from Iran, Egypt, Thailand, South Korea, China, Malaysia, and Belgium. In France, the same work performed on diarrheal dairy calves identified *C*. *parvum* as the persisting species causing infection in cattle [23]. Further, this species has been found to affect humans, thus affirming the observation that cattle on farms may serve as the reservoir for potential zoonotic transmission. For instance, in a study involving 14 children with diarrhea in Accra, Ghana, most (10) were found with *C*. *parvum* as one of the leading causes of diarrhea among these children [59]. Furthermore, another study in Ghana reported the presence of *C*. *parvum* and *C*. *hominis* in children below 14 years of age after screening 2232 participants [60]. Also, Redlinger and colleagues found the same *C*. *parvum* species as the main parasite affecting humans at the USA-Mexico border, though most cases were asymptomatic [61]. However, they observed severe implications for children under five years of age. Finally, in Portugal, Lobo and colleagues found the same *C*. *parvum* in raw water suspected of having been polluted with sewage and run-off from farms [61].

This study showed that treated water from taps did not contain *Cryptosporidium species*. This outcome could be due to the efficient filtration system to remove the parasite from the water during treatment. However, the wells in Atua and Akwernor were found to contain *Cryptosporidium sp*., which puts these areas at risk of infection since these wells serve as drinking water sources for some people. In Ghana, a study on sachet drinking water in Accra reported potential transmission threats of enteric pathogenic protozoan organisms, including *Cryptosporidium* species [51]. Also, a case study on drinking water in Italy reported contamination by *Cryptosporidium* oocyst [48]. In addition, they reported the presence of a *Cryptosporidium* genotype associated with wild animals in the river and tap water. These outcomes show that this persistent parasite threatens global public health since it can, to some degree, escape the water treatment.

In addition to *Cryptosporidium* sp., other intestinal parasites were observed in this study. These included *Giardia sp*., *Ascaris sp*., *Strongyloides sp*., *T*. *erichiun*, and *Paragorinus sp*., with a few observed mixed infections. In Uganda, a study conducted among children with diarrhea reported the prevalence of protozoa and helminth as 20.9% and 13.9% [62]. *Giardia lamblia* had the highest prevalence at 15.4%, followed by hookworm at nine percent (9%). All other intestinal parasites like *Entamoeba histolytica*, *Cryptosporidium* species, *Entamoeba coli*, *Isospora species*, *Ascaris lumbricoides*, *Hymenolepis nana*, *Schistosoma mansoni*, *Trichuris trichiura*, *Strongyloides stercoralis*, and *Taenia* species had a prevalence ranging from 2.5 to 5% [62]. In another study conducted in Senegal among preschool and school-aged groups, polyparasitism was observed with the different intestinal parasites present [63].

From these observations, the presence of *Cryptosporidium sp*. has public health implications for the human population, especially the younger age group, since they risk getting infected [56, 64]. Not only does this infection affect people’s health, but it also affects the parents’ financial status due to the cost incurred for treatment [65, 66]. Also, affected children experience a more significant disease burden, especially with inconsistency in school; thus, absenteeism. More importantly, the absence of medication to cure cryptosporidiosis worsens the cases of infection, which may lead to infant mortality [67].

## Conclusions

*Cryptosporidium sp*. was detected in cattle fecal matter and well water in the study communities. PCR analysis showed a higher prevalence of *Cryptosporidium sp*. in cattle, 86 (47.8%), than the prevalence of the other intestinal parasites. The study observed a higher intensity of the parasites in young animals than in adults; therefore, the young are the primary source of environmental contamination. The genetic sequencing and phylogenetic analysis confirmed *Cryptosporidium parvum* as the persistent species in the study area compared with sequences from the GenBank: HQ651731 from Iran, AB513881 from Egypt, JX948123 from Thailand, JX914623 from South Korea, KF128753 from China HQ540685 from Malaysia and KF944365 from Belgium. These are the species found to affect humans and, as such, confirm the cattle as the reservoir host for zoonotic transmission.

## Supporting information

S1 Fig Details of protozoa *Cryptosporidium* DNA sequence 62_CAin. The details of the 62_Cain *Cryptosporidium* DNA sequence are shown in Fig 10a; it is 435 bp, with molecular weights for the single and double-stranded DNA being 131.13 and 263.12 kD, respectively. It comprises 35.4% A, 12.6% C, 17.7% G, and 34.3% T. The percentage GC is 30.34%.
(DOCX)

S2 Fig Details of protozoa *Cryptosporidium* DNA sequence 92_CAIn. The details of the 92_Cain *Cryptosporidium* DNA sequence are shown in Fig 10b; it is 436 bp, with molecular weights for the single and double-stranded DNA being 131.43 and 263.60 kD, respectively. It comprises 35.8% A, 12.4% C, 17.7% G, and 34.2% T. The percentage GC is 30.05%.
(DOCX)

S3 Fig Details of protozoa *Cryptosporidium* DNA sequence 102_CAIn. The details of the 102_Cain *Cryptosporidium* DNA sequence are shown in Fig 10c; it is 440 bp, with molecular weights for the single and double-stranded DNA being 132.64 and 266.03 kD, respectively. It comprises 35.7% A, 12.7% C, 17.5% G, and 34.1% T. The percentage GC is 30.23%.
(DOCX)
