# Supplementary material for: Molecular epidemiology of Cryptosporidium species in Kpong and its environs, Ghana
Source: PLoS One. 2023 Feb 24;18(2):e0281216. doi: 10.1371/journal.pone.0281216 (PMC9956599; doi:10.1371/journal.pone.0281216)
Supplement: S1 Fig — The details of the 62_Cain Cryptosporidium DNA sequence are shown in Fig 10a; it is 435 bp, with molecular weights for the single and double-stranded DNA being 131.13 and 263.12 kD, respectively. It comprises 35.4% A, 12.6% C, 17.7% G, and 34.3% T. The percentage GC is 30.34%. (DOCX) [file pone.0281216.s001.docx]

# **Supporting information 1**

1 AAGCTCGTAG TTGGATTTCT GTTAATAATT TATAAAAAAT ATTTTGATGA ATATTTATAT

61 AATATTAACA TAATTCATAT TACTATATAT TTTAGTATAT GAAATTTTAC TTTGAGAAAA

121 TTAGAGTGCT TAAAGCAGGC ATATGCCTTG AATACTCCAG CATGGAATAA TATTAAAGAT

181 TTTTATCTTT CTTATTGGTT CTAAGATAAG AATAATGATT AATAGGGACA GTTGGGGGCA

241 TTTGTATTTA ACAGTCAGAG GTGAAATTCT TAGATTTGTT AAAGACAAAC TAATGCGAAA

301 GCATTTGCCA AGGATGTTTT CATTAATCAA GAACGAAAGT TAGGGGATCG AAGACGATCA

361 GATACCGTCG TAGTCTTAAC CATAAACTAG CCAAAC-AGA GATTGGAGGT TGTTCCTTAC

421 TCCTTCAGCA CCTTAA

**S1 Fig. Details of Protozoa *Cryptosporidium* DNA sequence 62_CAin.** The details of the 62_Cain *Cryptosporidium* DNA sequence are shown in Fig. 10a; it is 435 bp, with molecular weights for the single and double-stranded DNA being 131.13 and 263.12 kD, respectively. It comprises 35.4% A, 12.6% C, 17.7% G, and 34.3% T. The percentage GC is 30.34%.
