# Supplementary material for: Molecular epidemiology of Cryptosporidium species in Kpong and its environs, Ghana
Source: PLoS One. 2023 Feb 24;18(2):e0281216. doi: 10.1371/journal.pone.0281216 (PMC9956599; doi:10.1371/journal.pone.0281216)
Supplement: S2 Fig — The details of the 92_Cain Cryptosporidium DNA sequence are shown in Fig 10b; it is 436 bp, with molecular weights for the single and double-stranded DNA being 131.43 and 263.60 kD, respectively. It comprises 35.8% A, 12.4% C, 17.7% G, and 34.2% T. The percentage GC is 30.05%. (DOCX) [file pone.0281216.s002.docx]

# **Supporting information 2**

1 AAGCTCGTAG TTGGATTTCT GTTAATAATT TATAAAAAAA AATTTTAGAT GAATATTTAT

61 ATAATATTAA CATAATTCAT ATTACTATAT ATTTTAGTAT ATGAAATTTT ACTTTGAGAA

121 AATTAGAGTG CTTAAAGCAG GCATATGCCT TGAATACTCC AGCATGGAAT AATATTAAAG

181 ATTTTTATCT TTCTTATTGG TTCTAAGATA AGAATAATGA TTAATAGGGA CAGTTGGGGG

241 CATTTGTATT TAACAGTCGA GGTGAAATTC TTAGATTTGT TAAAGACAAA CTAATGCGAA

301 AGCATTTGCC AAGGATGTTT TCATTAATCA AGAACGAAAG TTAGGGGATC GAAGACGATC

361 AGATACCGTC GTAGTCTTAA CCATAAACTA TGCCAAATAG AGAT-GGAGG TTGTTCCTTA

421 CTCCTTCAGC ACCTTAA

**S2 Fig. Details of Protozoa *Cryptosporidium* DNA sequence 92_CAIn.** The details of the 92_Cain *Cryptosporidium* DNA sequence are shown in Fig. 10b; it is 436 bp, with molecular weights for the single and double-stranded DNA being 131.43 and 263.60 kD, respectively. It comprises 35.8% A, 12.4% C, 17.7% G, and 34.2% T. The percentage GC is 30.05%.
