# Supplementary material for: Molecular epidemiology of Cryptosporidium species in Kpong and its environs, Ghana
Source: PLoS One. 2023 Feb 24;18(2):e0281216. doi: 10.1371/journal.pone.0281216 (PMC9956599; doi:10.1371/journal.pone.0281216)
Supplement: S3 Fig — The details of the 102_Cain Cryptosporidium DNA sequence are shown in Fig 10c; it is 440 bp, with molecular weights for the single and double-stranded DNA being 132.64 and 266.03 kD, respectively. It comprises 35.7% A, 12.7% C, 17.5% G, and 34.1% T. The percentage GC is 30.23%. (DOCX) [file pone.0281216.s003.docx]

# **Supporting information 3**

1 AGCTCGTAGT TGGATTTCTG TTAATAATTT ATAAAAAATA TTTTGATGAA TATTTATATA

61 ATATTAACAT AATTCATATT ACTATATATT TTAGTATATG AAATTTTACT TTGAGAAAAT

121 TAGAGTGCTT AAAGCAGGCA TATGCCTTGA ATACTCCAGC ATGGAATAAT ATTAAAGATT

181 TTTATCTTTC TTATTGGTTC TAAGATAAGA ATAATGATTA ATAGGGACAG TTGGGGGCAT

241 TTGTATTTAA CAGTCAGAGG TGAAATTCTT AGATTTGTTA AAGACAAACT AATGCGAAAG

301 CATTTGCCAA GGATGTTTTC ATTAATCAAG AACGAAAGTT TAGGGGATCG AAGACGATCA

361 GATACCGTCG TAGTCTTAAC CATAAAGCTA TGCCCAAAAA AC-AAGAATG GAGGTTGTTC

421 CTTACTCCTT CAGCACCTTA A

**S3 Fig. Details of Protozoa *Cryptosporidium* DNA sequence 102_CAIn.** The details of the 102_Cain *Cryptosporidium* DNA sequence are shown in Fig. 10c; it is 440 bp, with molecular weights for the single and double-stranded DNA being 132.64 and 266.03 kD, respectively. It comprises 35.7% A, 12.7% C, 17.5% G, and 34.1% T. The percentage GC is 30.23%.
